# Influence of Mn, Fe, Co, and Cu Doping on the Photoelectric Properties of 1T HfS_2_ Crystals

**DOI:** 10.3390/ma15010173

**Published:** 2021-12-27

**Authors:** Der-Yuh Lin, Yu-Tai Shih, Wei-Chan Tseng, Chia-Feng Lin, Hone-Zern Chen

**Affiliations:** 1 Department of Electronic Engineering, National Changhua University of Education, Changhua 500208, Taiwan; dylin@cc.ncue.edu.tw; 2 Department of Physics, National Changhua University of Education, Changhua 500207, Taiwan; 3 Graduate Institute of Photonics, National Changhua University of Education, Changhua 500207, Taiwan; m0823007@gm.ncue.edu.tw; 4 Department of Materials Science and Engineering, National Chung Hsing University, Taichung 402202, Taiwan; cflin@dragon.nchu.edu.tw; 5 Department of Electronic Engineering, Hsiuping University of Science and Technology, Taichung 412406, Taiwan; hzc@hust.edu.tw

**Keywords:** doping, transition-metal dichalcogenides, photoelectric properties, HfS_2_, chemical vapor transport method, 1T phase, bandgap, activation energy, photoresponsivity

## Abstract

Doping plays a vital role in the application of transition-metal dichalcogenides (TMDCs) because it can increase the functionality of TMDCs by tuning their native characteristics. In this study, the influence of Mn, Fe, Co, and Cu doping on the photoelectric properties of HfS_2_ was investigated. Pristine, Mn-, Fe-, Co-, and Cu-doped HfS_2_ crystals were grown using the chemical vapor transport method. Scanning electron microscopy images showed that the crystals were layered and transmission electron microscopy, X-ray diffraction, and Raman spectroscopy measurements confirmed that the crystals were in the 1T-phase with a CdI_2_-like structure. The bandgap of pristine HfS_2_ obtained from the absorption and photoconductivity spectra was approximately 1.99 eV. As the dopant changed from Mn, Fe, and Co, to Cu, the bandgap gradually increased. The activation energies of the samples were determined using temperature-dependent current-voltage curves. After doping, the activation energy decreased, and the Co-doped HfS_2_ exhibited the smallest activation energy. Time-resolved photoresponse measurements showed that doping improved the response of HfS_2_ to light; the Co-doped HfS_2_ exhibited the best response. The photoresponsivity of HfS_2_ as a function of the laser power and bias voltage was measured. After doping, the photoresponsivity increased markedly; the Co-doped HfS_2_ exhibited the highest photoresponsivity. All the experimental results indicated that doping with Mn, Fe, Co, and Cu significantly improved the photoresponsive performance of HfS_2_, of which Co-doped HfS_2_ had the best performance.

## 1. Introduction

Transition metal dichalcogenides (TMDCs) [1,2,3,4,5,6,7,8,9] refer to the compounds of transition metal (TM) elements from group IVB to group VIIB in the periodic table and chalcogen elements. They have the general chemical formula TX_2_, where T represents a TM atom, and X represents a chalcogen atom such as S, Se, or Te. TMDCs are layered materials in which one unit layer is composed of three atomic planes. One TM atomic plane and two chalcogen atomic planes form an X-T-X sandwich structure via strong covalent bonds, and the X-T-X monolayers are gathered together by van der Waals forces. Owing to the weak van der Waals forces, foreign atoms or molecules can be easily inserted between the X-T-X monolayers [2], and TMDCs can be exfoliated into structures with few monolayers [10] or graphene-like two-dimensional (2D) freestanding monolayers [11]. Such 2D TMDCs with atomic-scale thicknesses exhibit a direct bandgap and strong spin-orbit coupling. Thus, 2D TMDCs have unique mechanical, chemical, optical, and electrical properties and have promising applications as catalysts [8,12], energy storage devices, electronic devices, biosensors, piezoelectric devices, photonic devices, and gas sensors [6].

The most noticeable TMDCs are those of the group VIB TM elements Mo and W, such as MoS_2_, WS_2_, MoSe_2_, WSe_2_, and MoTe_2_. They have an appropriate bandgap value between 1 and 2 eV, and by changing the dimensionality, this bandgap can be tuned from indirect in bulk materials to direct in monolayers. Their unique properties are favorable for applications in optoelectronics, sensing, photovoltaics, and nanoelectronics [3,7,13]. For example, Radisavljevic et al. fabricated the first monolayer MoS_2_ field-effect transistor [14], and Splendiani et al. and Mak et al. discovered strong photoluminescence in MoS_2_ monolayers [15,16].

Furthermore, TMDCs of the group IVB TM elements Zr and Hf, such as ZrS_2_, HfS_2_, ZrSe_2_, and HfSe_2_, also show semiconductor characteristics, and related research has been conducted [17]. According to previous reports, HfS_2_ and ZrS_2_ bulk materials have indirect bandgaps; the bandgap of HfS_2_ is approximately 1.80–2.13 eV [18,19,20,21,22,23], and the bandgap of ZrS_2_ is approximately 1.68–1.78 eV [18,19,20,21,24,25]. The corresponding light wavelengths fall within the range of the visible and infrared regimes. Therefore, they may be suitable for various applications in optoelectronics, such as photodetectors and photovoltaic devices [17,25].

2D layered HfS_2_ crystals have recently attracted significant attention owing to their prospective properties [26]. Kaur et al. employed a solvent-assisted ultrasonification method to chemically exfoliate HfS_2_ crystals into single or multiple monolayers. The HfS_2_ nanosheets exhibited an indirect bandgap of 1.3 eV and held a high potential for applications in field-effect transistors [27]. Wang et al. grew an HfS_2_ monolayer on hexagonal boron nitride (h-BN) via chemical vapor deposition; photodetectors based on HfS_2_/h-BN heterostructures exhibited excellent sensing performance [28]. Li et al. used exfoliated HfS_2_ nanosheets to manufacture memory devices, which exhibited typical bipolar resistive switching behavior with a high switching voltage and a small ratio of high and low resistive states [29]. Yin et al. showed that HfS_2_ nanosheets exhibit excellent nonlinear optical absorption in broadband and have the potential for applications in ultrafast photonics [30]. Hoat et al. utilized density functional theory to theoretically investigate the electronic structure and optical properties of HfS_2_ monolayers under vertical strain. The application of vertical strains can remarkably adjust the bandgap, and an indirect-direct gap transition may occur with compressive strains [31].

In optoelectronic devices and field-effect transistors, the most widely studied TMDC is MoS_2_; however, several experiments have shown that the performance of HfS_2_ is not inferior to that of MoS_2_ [32,33,34,35,36]. In addition, theoretical calculations predicted that the room-temperature mobility of 2D HfS_2_ is 1833 cm^2^ V^−1^ s^−1^, which is much higher than the 340 cm^2^ V^−1^ s^−1^ value of 2D MoS_2_ [37]. Xu et al. studied the electronic and optoelectronic properties of a few-layered HfS_2_ phototransistor. This ultrathin HfS_2_ phototransistor had a very high on/off ratio ≈10^7^ and an ultrahigh photoresponsivity of over 890 AW^−1^ [34]. These results indicate that HfS_2_ has a high application potential and is worthy of further in-depth research.

Doping is the intentional introduction of foreign impurities into the host material. It plays an essential role in the research on TMDCs because it can increase the functionality of TMDCs by providing routes to tune their native characteristics. For example, Suh et al. established stable *p*-type conduction in MoS_2_, which had intrinsic *n*-type conduction, by substitutional Nb doping [38]. Zhang et al. doped Mn into a MoS_2_ monolayer on a graphene substrate and altered its band structure [39]. Gong et al. demonstrated that changing the Se concentration can fine-tune the optical bandgap of Se-doped MoS_2_ [40]. Mouri et al. demonstrated the tunability of the photoluminescence properties of MoS_2_ monolayers via chemical doping [41]. Li et al. reported that field-effect transistors based on an Fe-doped SnS_2_ monolayer exhibited high optoelectronic performance [42].

Theoretically, many calculations have been conducted to investigate the structural, electronic, thermoelectric, optical, and magnetic properties of doped HfS_2_. In these studies, the dopants were of group IIIA, VA, VIIA [43,44,45], TM [46,47,48,49], or lanthanide atoms [50]. All these studies showed that doping is an effective method of modulating the properties of HfS_2_, and the doped HfS_2_ may have significant potential applications in photocatalysts, and tunable electronic, optoelectronic, thermoelectric, magneto-optic, and spintronic devices.

Based on theoretical studies, it is interesting and essential to experimentally investigate the properties of doped HfS_2_. However, the influence of doping with TM atoms on the photoelectric properties of HfS_2_ crystals is not completely understood. Thus, in this study, pristine, Mn-, Fe-, Co-, and Cu-doped HfS_2_ crystals were grown using the chemical vapor transport (CVT) method, and their photoelectric properties were explored. All the experimental results indicated that doping with Mn, Fe, Co, and Cu could significantly improve the photoresponsive performance of HfS_2_ crystals. The Co-doped HfS_2_ crystal exhibited the best performance.

## 2. Materials and Methods

Pristine, Mn-, Fe-, Co- and Cu-doped HfS_2_ crystals were grown using the CVT method. First, Hf and S were weighed using an electronic balance to generate a Hf to S molar ratio of 1:2. Then, these quantities of Hf and S as well as 0.5 g of I_2_ used as a transport agent were placed into a quartz ampoule along with the doping elements Mn, Fe, Co, and Cu. The designed doping concentration was 2%. The quartz ampoule was evacuated to 1 × 10^−3^ Pa, sealed, and then placed in a three-zone furnace for 300 h. To obtain the best diffusion gradient for crystal growth, the temperatures at both ends of the quartz ampoule were set to 880 °C and 730 °C, respectively. The temperature gradient was approximately 5 °C/cm.

After the growth of the pristine, Mn-, Fe-, Co-, and Cu-doped HfS_2_ crystals, a JEOL JXA-8530F (Tokyo, Japan) field-emission electron probe micro-analyzer (FE-EPMA) was used to identify the chemical composition. The morphology of the crystals was characterized by a HITACHI S-4800 (Hitachi High-Tech-, Tokyo, Japan) field-emission scanning electron microscope (SEM). A JEOL JEM-3010 (Jeol, Tokyo, Japan) transmission electron microscope (TEM) was used to obtain crystal images. The crystal structures of the samples were examined using a Bruker D8 SSS (Bruker, Billerica, MA, USA) high-resolution X-ray diffractometer (XRD) with Cu Kα radiation (λ = 1.5418 Å). A Dongwoo Ramboss (DongWoo Optron, Gwangju-Si, Korea) micro Raman system equipped with a solid-state laser source was used for the Raman analysis. The wavelength of the incident laser beam was 532 nm. The photoluminescence (PL) spectra of the samples were collected using a HORIBA iHR550 (Horiba, Kyoto, Japan) spectrometer. The wavelength of the excitation laser beam was 532 nm.

For absorption and photoconductivity (PC) measurements, a 1/4 m monochromator (MKS, Irvine, CA, USA) equipped with a 130 W halogen lamp was used to produce monochromatic light with a wide photon energy range. A mechanical chopper was used to modulate continuous light from the monochromator into alternating light. For the absorption measurements, the frequency of the alternating incident light was 200 Hz. To detect the intensity of the transmitted light, a silicon photodetector (Thorlabs, Newton, NJ, USA) with an amplifier was placed on the back of the measured sample. The output signals of the photodetector were recorded by a dual-phase lock-in amplifier (Ametek, Berwyn, PA, USA) to suppress the noise signals. For the PC measurements, the frequency of the incident alternating light was 20 Hz. A stable bias voltage of 20 V applied to the measured sample was supplied by a Keithley 2400 SourceMeter (Tektronix, Beaverton, OR, USA). To obtain the photoresponsivity of the measured sample, the photocurrent was recorded using a dual-phase lock-in amplifier and then divided by the power of the incident light at each wavelength.

A solar simulator, which provided a stable irradiation intensity of 100 mW/cm^2^, was used as the light source to record the current-voltage (I-V) characteristics of the measured sample under dark and illumination conditions. In addition, a Keithley 2400 SourceMeter was used to apply a bias voltage to the measured sample and record the current. To construct the temperature-dependent I-V curves, a Janis Research CCS-250 system (Lake Shore Cryotronics, Westerville, OH, USA) was utilized; this system was equipped with a Model 32 B cryogenic thermometer controller (Cryogenic Control Systems, Rancho Santa Fe, CA, USA) to adjust the temperature of the measured sample within the range of 300 to 380 K.

For time-resolved photoresponse measurements, a 532 nm wavelength laser was controlled by an AFG-2225 function generator (GW Instek, New Taipei City, Taiwan) to apply on/off light modulation to the measured sample. First, a stable bias voltage of 100 V was applied to the measured sample using a Keithley 2410 SourceMeter (Tektronix, Beaverton, OR, USA). Then, the photocurrent signals were collected using a data acquisition device with a sampling frequency of 1 MHz.

A laser with a wavelength of 532 nm was used as the excitation source to measure the photoresponsivity of the measured sample as a function of the laser power and bias voltage. The laser light was modulated into alternating light with a frequency of 1 Hz using an AFG-2225 function generator. For the laser power-dependent photoresponsivity measurements, the laser power was adjusted using neutral-density filters. A stable bias voltage of 100 V was applied to the measured sample using a Keithley 2400 SourceMeter. The photocurrent was recorded using a dual-phase lock-in amplifier and divided by the laser power to obtain the photoresponsivity of the measured sample. For the measurements of the bias-dependent photoresponsivity, the laser power was set to 1.3 mW, and a Keithley 2400 SourceMeter was used to apply a bias voltage to the measured sample and record the induced current. The difference between the average currents under dark and illumination conditions was divided by the incident laser power to obtain the photoresponsivity of the measured sample.

## 3. Results and Discussion

An FE-EPMA was used to determine the chemical composition of the grown samples, and the results are listed in Table 1. Each value in Table 1 is an average value after multiple measurements; therefore, the sum of the atomic percentages of Hf, S and the dopant for each sample was not exactly equal to 100%. The atomic ratio of Hf to S in the pristine HfS_2_ crystal was approximately 1:2. The Mn-, Co-, and Cu-doped HfS_2_ crystals were S-rich, whereas the Fe-doped HfS_2_ crystal was Hf-rich. In the Mn-, Co-, and Cu-doped HfS_2_ crystals, the atomic percentages of the dopants were much higher than that in the Fe-doped HfS_2_ crystal. Theoretical calculations [46] showed that under S-rich conditions, it is energetically favorable and relatively easier to incorporate TM atoms in HfS_2_. The FE-EPMA results were consistent with this prediction.

**Figure 1:**
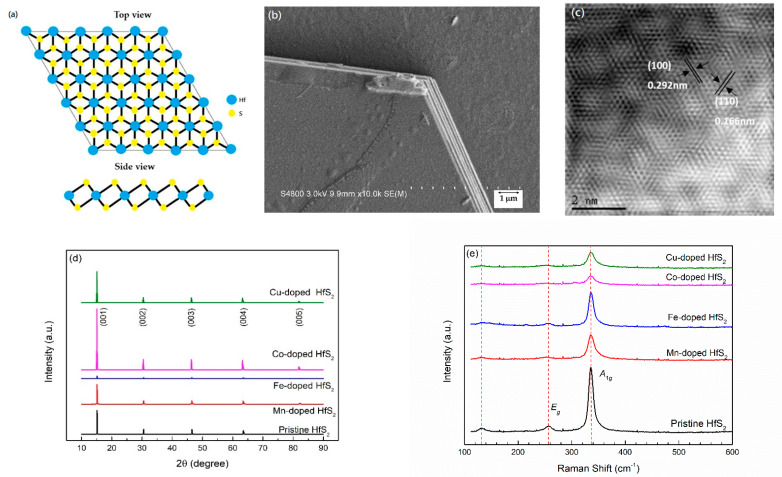
(**a**) Schematic structure of a 1T-HfS_2_ crystal; (**b**) Scanning electron microscopy image of the Cu-doped HfS_2_ crystal; (**c**) transmission electron microscopy image of the Co-doped HfS_2_ crystal; (**d**) X-ray diffraction patterns of the pristine, Mn-, Fe-, Co-, and Cu-doped HfS_2_ crystals; (**e**) Raman spectra of the pristine, Mn-, Fe-, Co-, and Cu-doped HfS_2_ crystals.

Figure 1a shows the schematic structure of a 1T-HfS_2_ crystal, and the top view and side view of layered forms are presented. In a HfS_2_ single layer, the Hf atomic plane is sandwiched between two S atomic planes. The Hf atom is octahedrally coordinated with the S atoms. 1T-HfS_2_ adopts a CdI_2_-like layered structure belonging to the space group $\bar{P}$3m1. Figure 1b shows an SEM image of the Cu-doped HfS_2_ sample. The grown HfS_2_ crystal was composed of multiple layers, and an angle of 120° characterized the edge of each layer. SEM images of the other samples are similar to that shown in Figure 1b.

Figure 1c shows a TEM image of the Co-doped HfS_2_ sample. This image confirms that the grown HfS_2_ crystal has a hexagonal 1T structure, and TEM images of the other samples are similar to that shown in Figure 1c. First, the TEM image of each HfS_2_ crystal was used to estimate the lattice plane spacings, *d*_100_ and *d*_110_. Then, the lattice constant *a* was calculated using the formula

(1) $$\frac{1}{d_{hkl}^{2}}=\frac{4}{3}\left(\frac{h^{2}+hk+k^{2}}{a^{2}}\right)+\frac{l^{2}}{c^{2}}.$$

The calculated results are listed in Table 2. It can be seen that as the dopant changed from Mn, Fe, and Co to Cu, the lattice spacings *d*_100,_ *d*_110_ and the lattice constant *a* gradually decreased. This reduction may be due to the substitution of doping atoms for Hf atoms. As the radius of the doping atoms decreased, *d*_100_, *d*_110_, and *a* also decreased.

XRD and Raman spectroscopy were used to confirm the crystal structures of the samples. Figure 1d shows the XRD patterns of the pristine, Mn-, Fe-, Co-, and Cu-doped HfS_2_ crystals. Only the (00*l*) diffraction peaks of the HfS_2_ crystals are observed in Figure 1d. The peaks at approximately 2θ = 15.16°, 30.52°, 46.54°, 63.48°, and 82.3° correspond to the (001), (002), (003), (004), and (005) planes of the HfS_2_ crystals, respectively. The diffraction patterns of the HfS_2_ crystals matched well with JCPDS card No. 28-0444, confirming that the crystals had a CdI_2_-like layered structure belonging to the space group $\bar{P}$3m1 [52]. As shown in Figure 1d, as the dopant of the HfS_2_ crystals changed from Mn, Fe, and Co to Cu, the position of the (001) peak shifted slightly to a smaller angle. Using the characteristic wavelength of the Kα radiation of copper λ = 1.5418 Å and Bragg’s formula

(2) 2dsinθ=nλ,

The lattice constant *c* of each HfS_2_ crystal was calculated and is listed in Table 2. The estimated *c* value of the pristine HfS_2_ crystal matched well with the value reported by Lucovsky et al. [51]. As shown in Table 2, as the dopant of the HfS_2_ crystals changed from Mn, Fe, and Co to Cu, the lattice constant *c* increased slightly; this small effect may be due to the doping of TM atoms, which reduces the lattice constant *a*, deforms the lattice, and then slightly increases the lattice constant *c*.

According to group theory, 1T-HfS_2_ has vibrational modes with symmetries of *A*_1g_ + *E_g_* + A_2*u*_ + *E*_u_ at the Γ point. The *A*_1g_ + *E_g_* modes are Raman-active, while the A_2*u*_ + *E*_u_ modes are infrared-active [21,53,54]. The Raman spectra of the pristine, Mn-, Fe-, Co-, and Cu-doped HfS_2_ crystals recorded at room temperature are shown in Figure 1e. There are weak peaks observed at approximately 131.7 cm^−1^. Neal et al. considered these peaks were due to the *E_u_*(TO) mode, which resulted from the in-plane out-of-phase vibrations of the atomic planes of S and the atomic planes of Hf [53]. However, the same *E_u_* mode was found in the infrared spectra at around 166 cm^−1^ [52] and 155 cm^−1^ [53]. Therefore, the origin of these weak peaks observed at approximately 131.7 cm^−1^ should still be further investigated. The weak peaks observed at approximately 256.2 cm^−1^ were due to the *E_g_* mode, which resulted from the in-plane out-of-phase vibrations of the atomic planes of S. The intense peaks observed at approximately 335.3 cm^−1^ were due to the *A*_1*g*_ mode, which resulted from the out-of-plane out-of-phase vibrations of the atomic planes of S [21,52,53,54,55]. The *E*_g_ and *A*_1g_ peaks are the two most commonly observed signals in 1T HfS_2_ crystals [21,27,53,55,56,57,58,59,60]. Figure 1e indicates that the grown HfS_2_ crystals were in the 1T phase. Furthermore, Figure 1e shows that the doping of Mn, Fe, Co, and Cu in the HfS_2_ crystals had no significant effect on the positions of the *E_g_*, and *A*_1*g*_ peaks.

**Figure 2:**
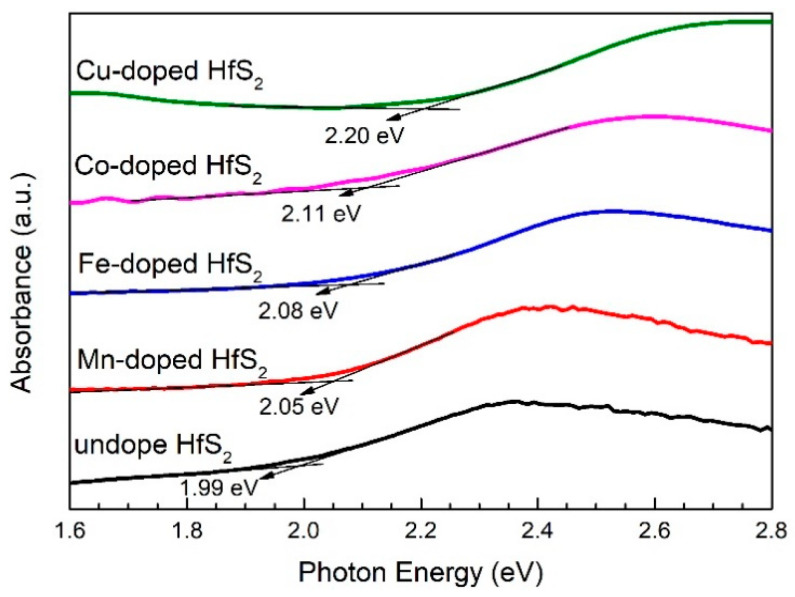
Absorption spectra of the pristine, Mn-, Fe-, Co-, and Cu-doped HfS_2_ crystals.

Figure 2 shows the absorption spectra of the pristine, Mn-, Fe-, Co-, and Cu-doped HfS_2_ crystals recorded at room temperature. From these spectra, the bandgap of each sample was determined. The bandgap of pristine HfS_2_ was approximately 1.99 eV, which is similar to the values reported by other researchers [18,19,20,21,22,23]. The bandgaps of the Mn-, Fe-, Co-, and Cu-doped HfS_2_ crystals were approximately 2.05, 2.08, 2.11, and 2.22 eV, respectively. As the dopant of the HfS_2_ crystals changed from Mn, Fe, and Co to Cu, the bandgap gradually increased. This increase may be a result of reducing the lattice spacings *d*_100_ and *d*_110_ and the lattice constant *a*.

**Figure 3:**
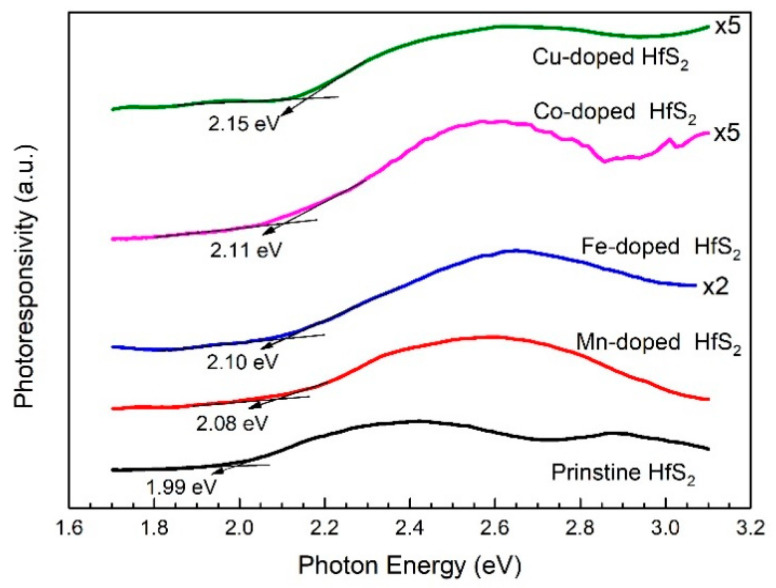
Photoconductivity spectra of the pristine, Mn-, Fe-, Co-, and Cu-doped HfS_2_ crystals.

The bandgaps of the HfS_2_ crystals could also be determined from their PC spectra. Figure 3 shows the PC spectra of the HfS_2_ crystals. The bandgaps of the pristine, Mn-, Fe-, Co-, and Cu-doped HfS_2_ crystals were approximately 1.99, 2.08, 2.10, 2.11, and 2.15 eV, respectively. These values were close to those specified by the absorption spectra.

**Figure 4:**
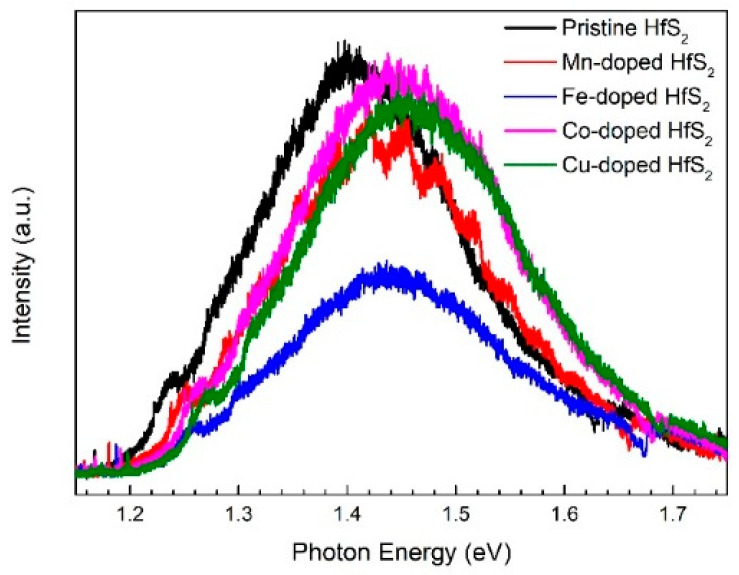
Photoluminescence spectra of the pristine, Mn-, Fe-, Co-, and Cu-doped HfS_2_ crystals.

Figure 4 shows the PL spectra of the HfS_2_ samples recorded at room temperature. The peaks of the spectra were located at 1.40~1.45 eV. Fu et al. recorded the PL spectra of HfS_2_ nanoflakes with a thickness of <1~5 nm [32]. The peaks of their spectra were located at approximately 1.45 eV. Fu et al. attributed the PL to the near-indirect bandgap emission of the HfS_2_ nanoflakes. Therefore, the PL from the samples in the present study may have resulted from the near-bandgap emission of the HfS_2_ nanoflakes in these samples. As the dopant changed from Mn, Fe, and Co to Cu, the PL peak blue-shifted; this shift may be due to an increase in the bandgap of the HfS_2_ nanoflakes in the samples.

**Figure 5:**
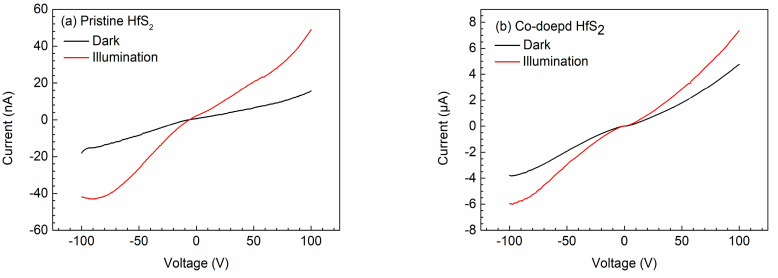
Current-voltage curves of (**a**) the pristine and (**b**) the Co-doped HfS_2_ crystals.

Figure 5 shows the I-V characteristic curves of the pristine and Co-doped HfS_2_ crystals with and without light illumination; the I-V curves of the other samples revealed similar behaviors. The resistivity of each sample was determined from the I–V curves and is listed in Table 3. As shown, doping with Mn, Fe, Co, and Cu can reduce the resistivity of HfS_2_ crystals. Notably, the Co-doped crystal exhibited the lowest resistivity, and when the HfS_2_ crystals were illuminated, their resistivity was considerably reduced.

**Figure 6:**
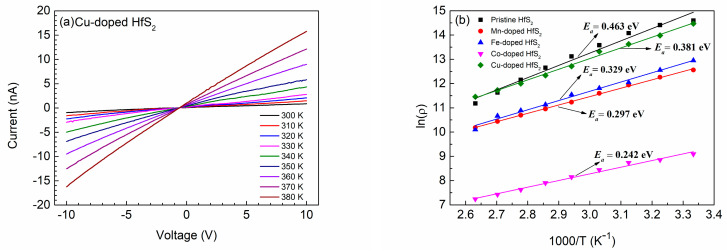
(**a**) Temperature-dependent I-V curves of the Cu-doped HfS_2_ crystal. (**b**) Plots of ln(*ρ*) versus 1000*/T* of the pristine, Mn-, Fe-, Co-, and Cu-doped HfS_2_ crystals.

To understand the relationship between the conductivity and temperature, I-V curves of each sample at various temperatures were constructed. Figure 6a shows the results for Cu-doped HfS_2_. Under a given bias voltage, the current of the Cu-doped HfS_2_ crystal increased as the temperature increased, and the temperature-dependent I-V curves of the other samples exhibited similar behaviors. This phenomenon resulted from the increase in carrier concentration with the increase in temperature, leading to a decrease in the resistivity of the samples.

For *n*-type semiconductors, the conduction process is mainly contributed by electrons that transition from the donor level to the conduction band. The relationship between resistivity *ρ* and temperature *T* can be expressed as [29,61,62,63]

(3) $$\rho \left(T\right)=\rho_{0}e^{E_{a}/k_{B}T},$$

where *ρ*_0_ is a constant, *k*_B_ is the Boltzmann constant, and *E_a_* is the activation energy, which approximately measures the energy difference between the bottom of the conduction band and the donor level. The lower the activation energy, the easier it is for the donor electrons to jump to the conduction band.

The resistivity *ρ* of each sample at various temperatures was determined from the temperature-dependent I-V curves. The curves of ln[*ρ* (*T*)] versus 1000/*T* for the samples are plotted in Figure 6b. As shown, the curve of each sample was approximately a straight line, and the Co-doped HfS_2_ crystal had the smallest *ρ* at any given temperature *T.* The activation energy of each sample was determined from the slope of each line in Figure 6b; the activation energies of the Mn-, Fe-, Co-, and Cu-doped HfS_2_ crystals were 0.297, 0.329, 0.242, and 0.381 eV, respectively. These values were all less than the activation energy of pristine HfS_2_, which was 0.463 eV. The Co-doped HfS_2_ crystal had the smallest activation energy; hence, its donor electrons were the easiest to transition to the conduction band.

**Figure 7:**
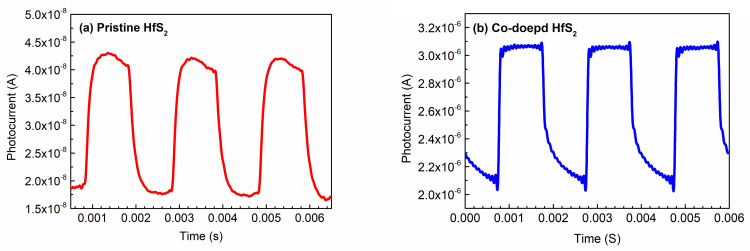
Photocurrents of (**a**) the pristine and (**b**) the Co-doped HfS_2_ crystals as a function of time under an illumination frequency of 500 Hz.

Figure 7 shows the time-resolved photoresponse measurements of the pristine and Co-doped HfS_2_ samples; this reveals how the photocurrent of each sample changed with time under an illumination frequency of 500 Hz. The photocurrents of the other samples exhibited similar behaviors under different illumination frequencies. Table 4 lists the rise time *t*_rise_ (from 10% to 90% of the maximum photocurrent) and the fall time *t*_fall_ (from 90% to 10% of the maximum photocurrent) of each sample under different illumination frequencies. As shown in Table 4, under any illumination frequency, the rise and fall times of the Mn-, Fe-, Co-, and Cu-doped HfS_2_ crystals were shorter than those of the pristine HfS_2_ crystal. The rise and fall times of the Co-doped HfS_2_ crystal were the shortest.

The current amplitude, which is defined as the difference between the maximum and minimum photocurrents in a rising-falling period, of each sample under different illumination frequencies, is listed in Table 5. Under any illumination frequency, the current amplitude of each TM-doped HfS_2_ crystal was greater than that of the pristine HfS_2_ crystal, and the Co-doped HfS_2_ crystal had the largest current amplitude. According to the data listed in Table 4 and Table 5, Mn, Fe, Co, and Cu doping significantly improved the response of HfS_2_ crystals to light. In particular, the Co-doped HfS_2_ crystal exhibited the best response to light.

**Figure 8:**
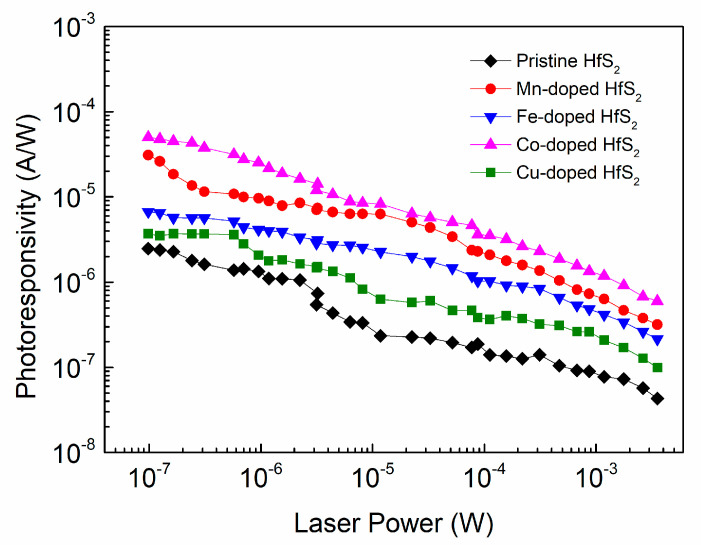
Photoresponsivity of the pristine, Mn-, Fe-, Co-, and Cu-doped HfS_2_ crystals as a function of laser power.

Figure 8 shows how the photoresponsivity of each sample varied with the incident laser power. As the laser power gradually decreased from the order of 10^−3^ W to the order of 10^−7^ W, the photoresponsivity of each sample gradually increased; this increase reached two orders of magnitude. For a given incident laser power, the photoresponsivity of each TM-doped HfS_2_ crystal was greater than that of the pristine HfS_2_ crystal, and the Co-doped HfS_2_ crystal exhibited the highest photoresponsivity. Its maximum value reached 49.96 μA/W at a laser power of 10^−7^ W, which is greater than the maximum photoresponsivity of the Mn-doped HfS_2_ crystal, 30.93 μA/W, and much greater than those of the pristine, Fe-, and Cu-doped HfS_2_ crystals.

**Figure 9:**
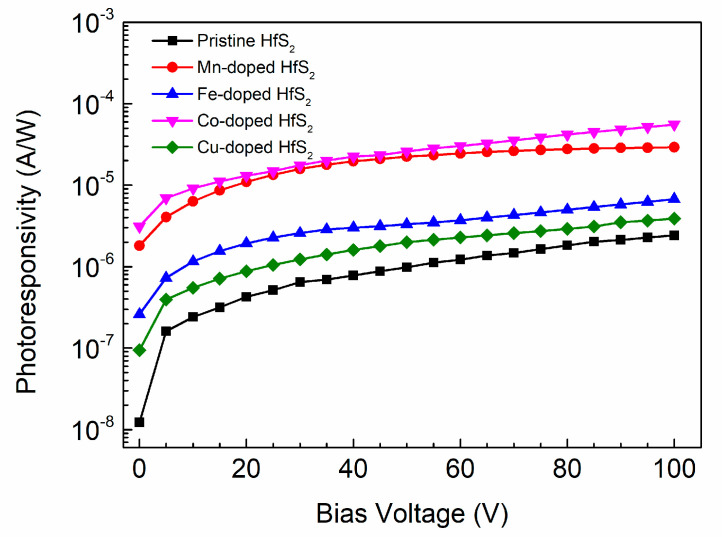
Photoresponsivity of the pristine, Mn-, Fe-, Co-, and Cu-doped HfS_2_ crystals as a function of bias voltage.

Figure 9 shows how the photoresponsivity of each sample varied with the bias voltage. As the applied bias voltage increased, the photoresponsivity of each sample also increased. For a given bias voltage, the photoresponsivity of each TM-doped HfS_2_ crystal was greater than that of the pristine HfS_2_ crystal, and the Co-doped HfS_2_ crystal exhibited the highest photoresponsivity. Its maximum value reached 55.70 μA/W at 100 V, which is greater than the maximum photoresponsivity of the Mn-doped HfS_2_ crystal, 29.16 μA/W, and much greater than those of the pristine, Fe-, and Cu-doped HfS_2_ crystals.

## 4. Conclusions

In conclusion, pristine, Mn-, Fe-, Co-, and Cu-doped HfS_2_ crystals were grown using the CVT method to study their structural, optical, and photoelectric properties. SEM images showed that the HfS_2_ crystals were layered materials, with an angle of 120° characterizing the edge of each layer. The TEM and XRD results showed that the HfS_2_ crystals had a CdI_2_-like 1T structure belonging to the space group $\bar{P}$3m1. As the dopant changed from Mn, Fe, and Co to Cu, the lattice constant *a* gradually decreased and the lattice constant *c* slightly increased. The signals of the *E_g_* and *A*_1*g*_ vibration modes in the Raman spectra also confirmed that the HfS_2_ crystals were in the 1T phase. Moreover, the bandgap of the pristine HfS_2_ crystal was determined as approximately 1.99 eV using the absorption and photoconductivity spectra. When the dopant was changed from Mn, Fe, and Co to Cu, the bandgap gradually increased. The PL peak from the HfS_2_ nanoflakes in the pristine sample was located at approximately 1.40 eV and blue-shifted as the dopant changed from Mn, Fe, and Co to Cu. The I-V curves revealed that Mn, Fe, Co, and Cu doping significantly increased the conductivity of HfS_2_; the Co-doped HfS_2_ crystal exhibited the highest conductivity. Light illumination also improved the conductivity of the samples. Furthermore, the activation energy of each HfS_2_ crystal was determined from the temperature-dependent I-V curves. After doping with Mn, Fe, Co, and Cu, the activation energy of HfS_2_ decreased, and the Co-doped HfS_2_ crystal had the lowest activation energy. The time-resolved photoresponse measurements revealed that Mn, Fe, Co, and Cu doping significantly improved the response of HfS_2_ to light. The Co-doped HfS_2_ crystal, which had the shortest rise and fall times and the largest current amplitude, exhibited the best response to light. Additionally, experiments on laser power-dependent and bias voltage-dependent photoresponsivity revealed that Mn, Fe, Co, and Cu doping increased the photoresponsivity of HfS_2_, of which the Co-doped HfS_2_ crystal exhibited the maximum photoresponsivity. Overall, doping with Mn, Fe, Co, and Cu significantly improved the photoresponsive performance of HfS_2_. In particular, the Co-doped HfS_2_ crystal exhibited the best photoresponsive performance. HfS_2_ crystals doped with Mn, Fe, Co, and Cu have tunable and excellent photoelectric properties, making them promising for use in sensing and photoelectronic devices.

## Figures and Tables

**Table 1:** Atomic percentages of Hf, S, and dopants in the pristine, Mn-, Fe-, Co-, and Cu-doped HfS_2_ crystals.

Sample	Hf (at. %)	S (at. %)	Dopant (at. %)
Pristine HfS_2_	34.26	65.75	
Mn-doped HfS_2_	31.44	68.00	0.54
Fe-doped HfS_2_	37.92	61.91	0.16
Co-doped HfS_2_	32.30	67.09	0.60
Cu-doped HfS_2_	30.78	68.24	0.96

**Table 2:** Lattice parameters of the pristine, Mn-, Fe-, Co-, and Cu-doped HfS_2_ crystals.

Sample	*d*_100_ (Å)	*d*_110_ (Å)	*a* (Å)	*c* (Å)
1T HfS_2_			3.63 ^1^	5.85 ^1^
Pristine HfS_2_	3.17	1.84	3.66	5.85
Mn-doped HfS_2_	3.09	1.79	3.57	5.86
Fe-doped HfS_2_	2.98	1.71	3.44	5.86
Co-doped HfS_2_	2.92	1.66	3.37	5.87
Cu-doped HfS_2_	2.82	1.59	3.26	5.87

^1^ Ref. [51].

**Table 3:** The resistivity of the pristine, Mn-, Fe-, Co-, and Cu-doped HfS_2_ crystals.

Sample	*ρ* (Ω⋅m)	
	Dark	Illumination
Pristine HfS_2_	2.184 × 10^6^	7.231 × 10^5^
Mn-doped HfS_2_	2.847 × 10^5^	1.791 × 10^5^
Fe-doped HfS_2_	4.211 × 10^5^	1.976 × 10^5^
Co-doped HfS_2_	8.898 × 10^3^	5.545 × 10^3^
Cu-doped HfS_2_	1.917 × 10^6^	1.389 × 10^6^

**Table 4:** Rise time *t*_rise_ and fall time *t*_fall_ of the pristine, Mn-, Fe-, Co-, and Cu-doped HfS_2_ crystals under different illumination frequencies.

	Frequency (Hz)							
	1		100		500		1000	
Sample	*t*_rise_ (ms)	*t*_fall_ (ms)	*t*_rise_ (ms)	*t*_fall_ (ms)	*t*_rise_ (μs)	*t*_fall_ (μs)	*t*_rise_ (μs)	*t*_fall_ (μs)
Pristine HfS_2_	13.20	17.44	0.28	0.66	175	264	123	132
Mn-doped HfS_2_	8.26	10.09	0.26	0.32	49	145	44	85
Fe-doped HfS_2_	8.71	11.46	0.29	0.38	53	157	48	93
Co-doped HfS_2_	7.84	9.41	0.24	0.29	43	121	40	62
Cu-doped HfS_2_	9.37	14.3	0.27	0.53	62	187	72	129

**Table 5:** Current amplitudes of the pristine, Mn-, Fe-, Co-, and Cu-doped HfS_2_ crystals under different illumination frequencies.

	Frequency (Hz)			
	1	100	500	1000
Sample	Current Amplitude (nA)			
Pristine HfS_2_	4.73	3.06	2.21	1.17
Mn-doped HfS_2_	818	712	630	450
Fe-doped HfS_2_	428	308	206	212
Co-doped HfS_2_	995	959	735	565
Cu-doped HfS_2_	97.2	68.1	50.0	46.6

## Data Availability

Data is contained within the article.
